# Use of facile mechanochemical method to functionalize carbon nanofibers with nanostructured polyaniline and their electrochemical capacitance

**DOI:** 10.1186/1556-276X-7-111

**Published:** 2012-02-08

**Authors:** Xusheng Du, Hong-Yuan Liu, Guipeng Cai, Yiu-Wing Mai, Avinash Baji

**Affiliations:** 1 Centre for Advanced Materials Technology (CAMT), School of Aerospace Mechanical and Mechatronic Engineering, J07 University of Sydney, Sydney, NSW 2006, Australia

**Keywords:** carbon nanofiber, conducting polymer, nanocomposite, polyaniline, capacitance, mechanochemistry

## Abstract

A facile approach to functionalize carbon nanofibers [CNFs] with nanostructured polyaniline was developed via *in situ *mechanochemical polymerization of polyaniline in the presence of chemically treated CNFs. The nanostructured polyaniline grafting on the CNF was mainly in a form of branched nanofibers as well as rough nanolayers. The good dispersibility and processability of the hybrid nanocomposite could be attributed to its overall nanostructure which enhanced its accessibility to the electrolyte. The mechanochemical oxidation polymerization was believed to be related to the strong Lewis acid characteristic of FeCl_3 _and the Lewis base characteristic of aniline. The growth mechanism of the hierarchical structured nanofibers was also discussed. After functionalization with the nanostructured polyaniline, the hybrid polyaniline/CNF composite showed an enhanced specific capacitance, which might be related to its hierarchical nanostructure and the interaction between the aromatic polyaniline molecules and the CNFs.

## Introduction

As a conducting polymer, polyaniline [PANI] has attracted much attention in recent years due to its potential applications in various hi-tech areas, for example, electrochemical displays, sensors, catalysis, capacitors, anti-corrosion coatings, electromagnetic shielding, and secondary batteries [1,2]. Many methods to prepare PANI have been developed [3-10], including interfacial polymerization, templates, and surfactant-assisted strategies, as well as mechanochemical methods. Since the mechanochemical methods have also been applied to disperse nanofillers in engineering polymers [11,12], it is worthwhile to extend this facile technique to modify functional conductive nanoparticles with PANI nanomaterials as the combination of PANI with electrically conductive nanoparticles (such as carbon nanotubes and graphite nanosheets) has been recently demonstrated to be a promising approach to improve their electronic or electrochemical performance [13-21].

As a one-dimensional carbon nanomaterial, carbon nanofibers [CNFs] are much easier to produce in large scale and are cheaper than the well-known carbon nanotubes [CNTs]. It is expected that the corresponding PANI/CNF composites have a wider range of useful properties and hence more promising commercial applications. However, a study on the functionalization of these cheap carbon nanomaterials with PANI is limited compared with the many investigations on PANI/CNT composites. In this paper, we described a simple route to the modification of CNF via *in situ *mechanochemical polymerization of PANI in the presence of chemically treated CNFs. It was found that the resultant composite was easy to disperse in ethanol and that the dispersion had very good stability, which is believed to be related to the novel hierarchical nanostructure formed during *in situ *mechanochemical polymerization. Moreover, the introduction of PANI greatly enhanced the electrochemical specific capacitance of CNFs.

## Experimental details

### Materials

CNFs (Pyrograf Products Inc., Cerdaville, OH, USA) were first treated with nitric acid to remove the metal impurities in the product. CNF modified with a PANI sample was prepared using the following procedure. In a typical process, 1 g aniline (98%; Sigma-Aldrich, New South Wales, Australia) and 0.2 g treated CNF powder were mixed and hand ground for 1 min in a 250 mL glass mortar in a glove box, and 5 g FeCl_3 _powder was then added in several portions in 10 min and mixed together with further grinding. After grinding for another 10 min, the product was collected and purified by washing with water and ethanol. A small portion of the wet product was then dispersed in 10 mL ethanol. The stability of the dispersion was studied, and some of its drops were also transferred to the copper grids for transmission electron microscopy [TEM] analysis. Pure PANI was also prepared without CNFs using the same mentioned procedure.

### Characterization and measurements

X-ray diffraction [XRD] patterns were obtained using an X-ray diffractometer (Siemens 5000, Siemens AG, Munich, Germany) with Ni-filtered Cu Kα radiation. Scanning electron microscopy [SEM] (Zeiss ULTRA plus, Carl Zeiss AG, Oberkochen, Germany) and TEM (Philips CM12, Philips, Eindhoven, The Netherlands) were used to examine the morphologies of the prepared samples. Fourier transform infrared [FT-IR] and UV-visible [vis] spectrum of the products were recorded on a Varian FT-IR spectrometer (Varian Inc., Palo Alto, CA, USA) and a Cary 5-UV-vis spectrometer (Agilent Technologies Inc., Santa Clara, CA, USA), respectively. Differential scanning calorimetry [DSC] data were obtained with a TA modulated DSC 2920 instrument at a heating rate of 20°C/min in nitrogen. Weight loss temperatures of the products were measured with a TA thermogravimetric analyzer (TGA 2950, TA Instruments, New Castle, DE, USA) at a heating rate of 20°C/min in N_2 _atmosphere. A CHI1202A Electrochemical Analyzer (CH Instruments, Austin, TX, USA) and a three-electrode electrochemical cell were used for electrochemical measurements. The sample was dispersed in 1 mL ethanol and sonicated for 20 min to prepare a suspension; 5 μL of such solution was added to the surface of the glass carbon electrode (*Φ *= 3 mm), followed by a drop of 5 wt.% Nafion solution to fix the material on the surface of the electrode. Prior to electrochemical measurements, the material-covered electrode was soaked in a 1-M H_2_SO_4 _solution for 2 min. The counter electrode was a Pt foil, and the reference electrode was a saturated calomel electrode.

## Results and discussion

**Figure 1:**
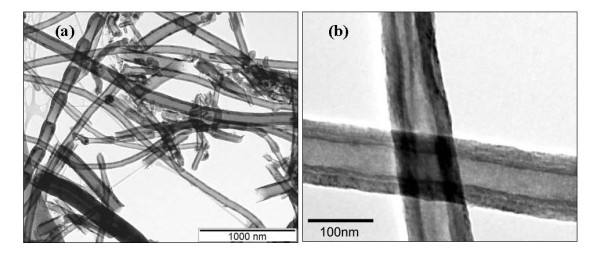
**TEM image of CNF samples with (a) low and (b) high magnifications**.

Figure 1 shows the TEM images of CNFs, which reveal that the samples contain mainly tube-like carbon nanomaterials with an outer diameter in the range of 50 to 150 nm and length of up to several microns with a few impurities, including a few bamboo-like nanofibers and spherical nanoparticles. These impurities usually appear in the vapor-grown carbon nanomaterials. The term 'CNFs' was named by the manufacturer, most likely to avoid confusion with conventional CNTs (whose diameters are much less than 100 nm).

**Figure 2:**
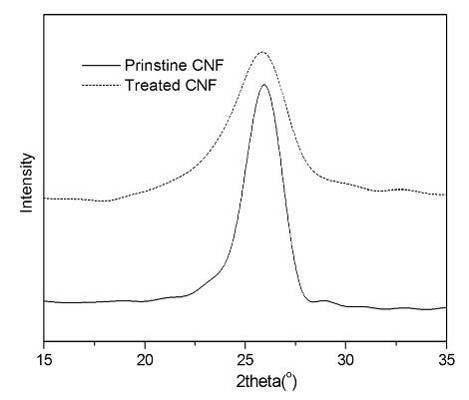
**XRD pattern of CNF samples**.

**Figure 3:**
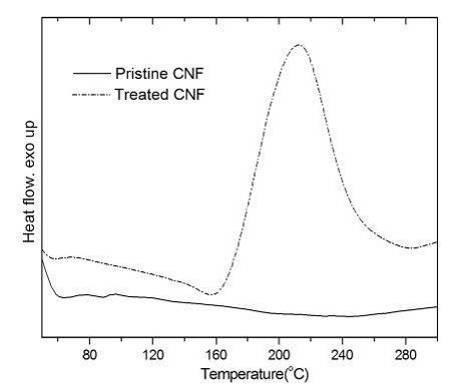
**DSC curves of CNF samples**.

In order to remove the metal impurities in the carbon nanomaterials and enhance the affinity of graphite to other foreign molecules, chemical treatment of the graphite is often needed before preparing the polymer nanocomposites. Pristine CNFs show only one diffraction peak around 2*q *= 25.9° (Figure 2), which represents the interlayer spacing of graphene layers in the sample. Chemical treatment results in a slightly reduced intensity of the diffraction peak, as shown in Figure 2. DSC analyses confirm the existence of organic groups on the chemically treated CNFs. As shown in Figure 3, in contrast to the plain curve of pristine CNFs, a strong exothermal peak appears around 210°C in the DSC curve of treated CNFs, which is due to the decomposition of organic groups, similar to the thermal behavior of graphite oxide in the same temperature range [22]. In the derivative thermogravimetry curve of the functionalized CNFs [Figure S1 in Additional file 1], two major stages of weight loss can be observed. The weight loss below 100°C can be attributed to the loss of free water, and the weight loss around 210°C is from the decomposition of functional groups on the surface of the CNFs, consistent with the mentioned DSC analysis.

**Figure 4:**
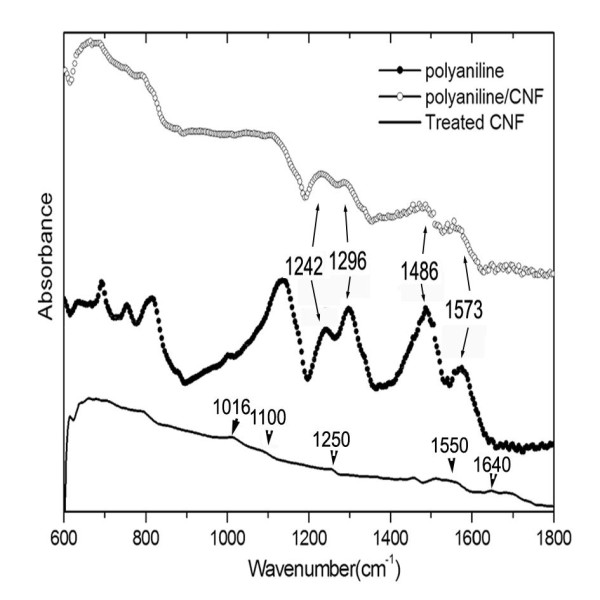
**FTIR-ATR spectrum of the chemically treated CNF, PANI/CNF, and PANI**.

Carbon materials usually show very weak IR response, and the baselines of their spectra are often slanted. In the FT-IR spectrum of the nitric acid-treated CNFs (Figure 4), the detectable broad peaks at 1,016, 1,100, 1,250, 1,550, and 1,640 cm^-1 ^suggest the existence of some functional organic groups, such as C-O, C-OR, C = O, and carboxyl groups, which could facilitate the adsorption of aniline via hydrogen bond. The FT-IR spectrum of the PANI displays characteristic absorption bands of PANI at 1,573, 1,486, 1,296, and 1,242 cm^-1^, which can be attributed to the C = C stretching vibration mode of the quinonoid and benzenoid rings, the stretching mode of C-N and the protonated C-N group, respectively [9]. PANI/CNF is expected to be richer in quinonoid units than pure PANI as aromatic structures are known to interact strongly with the graphitic surface via pi stacking. Indeed, in the spectrum of the composite, it is seen that the intensity of the band at 1,573 cm^-1 ^relative to that at 1,486 cm^-1 ^is increased. Figure 4 also shows that the intensities of most peaks of the composite are more decreased compared with those of the PANI, indicating that good adhesion of the polymer to CNFs leads to restricted motion of the PANI macromolecular chains. These interactions may facilitate the charge transfer process between PANI and CNFs, and increase the effective degree of electron delocalization.

**Figure 5:**
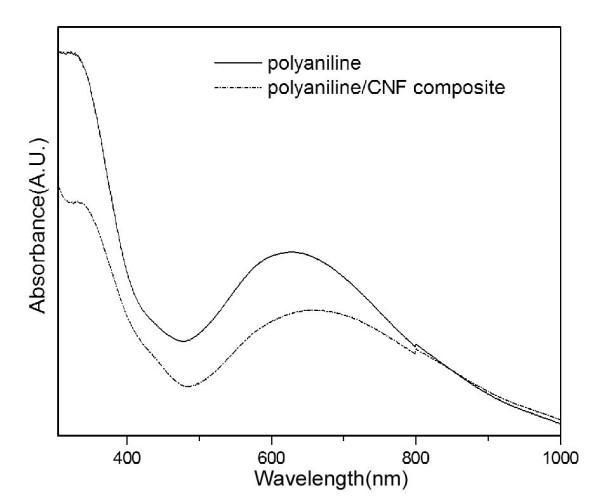
**UV-vis spectra of PANI and PANI/CNF composites**.

The UV-vis spectrum of PANI (Figure 5) shows characteristic absorption peaks of PANI in its emeraldine base [EB] form, where a narrow peak at 323 nm and broad peak at 620 nm occur, corresponding to the ***π***→π* transition centered on the benzenoid unit of EB and the quinonoid excitation band, respectively. The PANI peak at 620 nm is shifted to 660 nm after the formation of the PANI/CNF composite. A similar redshift was also observed in the previous report on PANI/graphene composites [23] and can be caused by the interaction between the aromatic polyaniline macromolecules and graphenes in CNFs.

**Figure 6:**
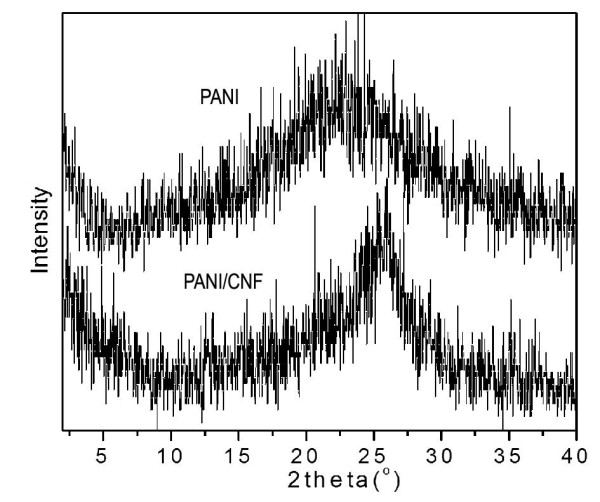
**XRD pattern of PANI and PANI/CNF composites**.

In the XRD pattern of PANI (Figure 6), one broad peak around 2*θ *= approximately 21° can be observed, which is the typical amorphous scattering of PANI in the EB form [24]. However, a stronger broad peak around 2*θ *= approximately 25.6° appears in the pattern of the PANI/CNF composite. As the CNFs also show a peak in the same 2*θ *range (Figure 2), the peak seen here could be due to the superimposition of peaks of EB-formed PANI and CNFs. It should be pointed out that the dendritic polyaniline nanofibers prepared using solid state mechanochemical polymerization with aniline hydrochloride as reagent were in the highly doped emeraldine salt form with high crystallinity [9,10]. However, the reactant used here is aniline, which may lead to different emeraldine form of the product. Clearly, detailed mechanochemical reaction mechanism needs further investigation.

**Figure 7:**
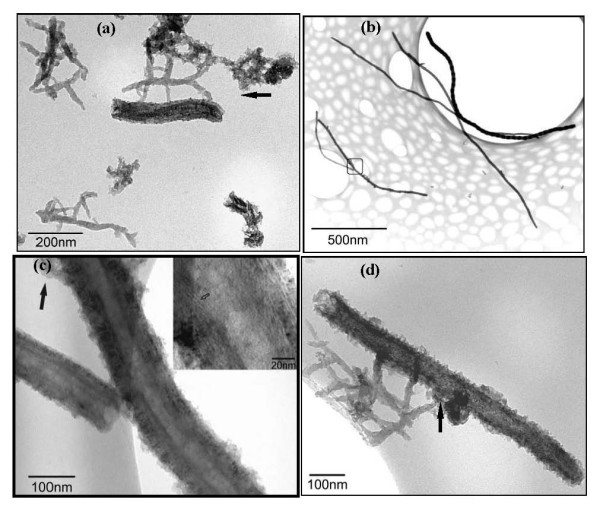
**TEM image of PANI/CNF composite**. PANI/CNF composite with different nanostructure (**a**) PANI branched nanofibers, (**b**, **c**) core shell composites and (**d**) PANI branched fibers grafted onto CNF.

In the TEM images of the composite, two main types of structures can be observed, e.g., branched PANI nanofibers in Figure 7a and new nanostructures that consist of CNFs modified by nanostructured blocks of PANI (Figure 7b, c, d). The branched nanofibers with diameters at approximately 40 nm and lengths of several hundred nanometers in Figure 7a are typical products of the solid state mechanochemical polymerization of PANI [9,10]. However, CNFs modified with PANI show two main morphologies. Figure 7b, c reveals a core shell structure in which the CNF is wrapped by a PANI nanolayer. There is a clearly visible tube-like carbon nanofiber core and a PANI shell in Figure 7c. Although the coating layers seem uniform and cover the entire surface of CNFs in the TEM image at lower magnification (Figure 7b), they are very rough, and many small protuberances appear on the surface of CNFs in higher magnification (Figure 7c, d), which contrast the smooth surface of CNFs (Figure 1b). The thickness of the coating is several tens of nanometers. The inset high-resolution TEM in Figure 7c shows that the tube wall of CNFs consists of stacked angled graphenes (as highlighted by the unfilled arrow in the inset figure) hence confirming that the so-called 'stacked cup' or 'herring bone' structures [25] remain after the functionalization of the polymer. Another main morphology of PANI combined with CNFs shows some thicker blocks or branched nanofibers of PANI grafted on the CNFs. The black arrows in Figure 7 highlight such blocks, which may have resulted from the secondary growth of the small blocks or protuberances of PANI on CNFs (Figure 7c, d) [10]. Such PANI blocks can also be observed in the SEM images, as highlighted by the blue arrows in Figure S2 in Additional file 1.

**Figure 8:**
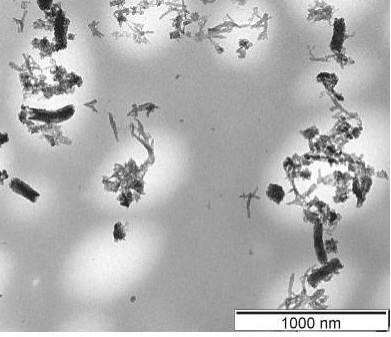
**TEM image of PANI/CNF composite in the ethanol suspension after centrifugation of 1,3000 rpm**.

The entirely hierarchical structure confers upon these functionalized CNFs' good dispersibility and stability of the dispersion, despite the much larger diameter and size of the CNF scaffolds than those of normal single-walled or multiwalled CNTs [MWCNTs]. The composite contains approximately 50 wt.% CNFs as calculated by the mass ratio between CNFs used and the PANI/CNF composite product. The composite particles readily dispersed in ethanol by ultrasonic irradiation, and the majority of them still remained dispersed in the solvent even after 5 min of centrifugation at 13,000 rpm. TEM of the upper part of the centrifuged solution shows both branched PANI nanofibers and CNFs modified with the nanostructured PANI (Figure 8). It is noted that the length of CNFs in this part is always less than a micron, indicating the dispersion of the composites prepared with shorter CNFs is more stable. Additionally, it is noted that the PANI adhered very strongly on the surface of the CNFs such that some polymer blocks were still found on the CNFs in the TEM images even after their solution was subjected to 20 min of ultrasonic irradiation.

The main reason for good dispersibility of these novel functionalized CNF composites is believed to come from their overall nanostructured morphology. Both components, the PANI and the CNFs modified with the PANI nanostructures, become stable in dispersion because of their nanometric sizes and the stereo-hindrance effect of the nanostructured PANI. Ethanol dispersions of PANI dendritic nanofibers are very stable due to their highly branched nanostructure as reported recently [10]; hence, it is believed that the mechanism of stabilization for the CNFs functionalized with PANI is the same, and the CNFs behave like scaffolds for PANI. Also, the rapid formation of homogeneous and stable dispersions with ultrasonic irradiation may contribute to the good segregation of the material to its individual nanostructure. The direct preparation and stability of the dispersions represent a significant advancement in processing PANI/CNF composites. All the obtained results show that the nanostructure of PANI has lead to enhanced processability of these composites. In a previous work [21], PANI/graphite nanosheet composites were synthesized, and fine polyaniline particles adhered on the surfaces of graphite nanosheets. Due to the large size of the graphite nanosheet (up to 20 μm in diameter), the dispersion of the composites is very unstable and ready to precipitate. This indicates the small size of the CNFs also contributes to the stability of the dispersions, as shown in Figure 8.

**Figure 9:**
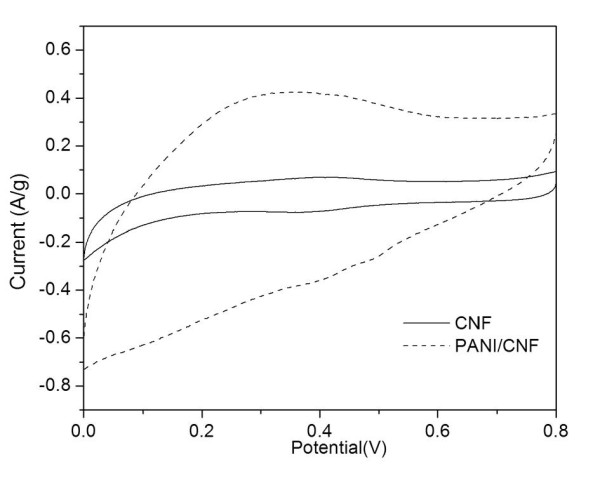
**Cyclic voltammograms**. PANI/CNF composite and CNF samples in 1 M H_2_SO_4 _aqueous solution with a scan rate of 2 mV/s.

The electrochemical activity of the products was investigated by the cyclic voltammetry [CV] method. As shown in Figure 9, the oxidation process of polyaniline with a peak at approximately 0.25 V is caused by oxidation of leucoemeraldine to the emeraldine form [26]. The CV of the composite is similar to that of the PANI/MWCNT reported recently [14,15]. These results indicate that the PANI/CNF composite is electroactive. In contrast, the CNF sample exhibits only the capacitance current, and the current is much lower than that of the PANI/CNF composite. From the CV, we can calculate the specific capacitance of the samples, according to Yang et al. [27]:

(1) $$C=\frac{\int I\cdot dV}{v\cdot m\cdot \Delta V},$$

where *I *is the response current (amperes); Δ*V *is the potential window (volts); *v *is the potential scan rate (volts per second), and *m *is the mass of electrode materials (grams). According to Figure 9, PANI/CNF has a specific capacitance of 139.8 F/g, which is less than that of PANI/MWCNT (approximately 190 F/g with the same PANI loading) at the same scanning rate [14]. However, the specific capacitance of the PANI/CNF composite is much higher than that of the CNF (22 F/g as calculated from Figure 9), thus highlighting the remarkable enhancement of the capacitance of CNFs by modification of PANI via the mechanochemical polymerization method. The enhancement (approximately six times) of the capacitance of CNF by the modification of PANI is comparable to that of MWCNTs modified by *in situ *polymerization of PANI [14]. The cyclic stability of the composite was also studied. As shown in Figure S3 in Additional file 1, the current (for instance, at 0.45 V) still retains approximately 93% of the original value after 500 cycles, indicating a good electrochemical stability of the composite. Considering the lower price and availability of CNFs, they could find more promising commercial applications.

At present, the growth mechanism of mechanochemical polymerization of polyaniline nanostructures remains unclear, and further investigations are required. Some may argue that polymerization did not take place during the mechanical processing but occurred after the samples were purified with water. However, this is unlikely the case since it is convenient to monitor the process of polymerization by the obvious appearance change of the mixture. The reaction proceeded so quickly that the loose mixture of the powders of CNF and aniline would change to a hard block in just 1 min when all the FeCl_3 _was added in one portion. In contrast, when aniline was polymerized with FeCl_3 _as an oxidation agent in water, no polyaniline products precipitated in such a short time, and even the color of the solution did not change. All these indicated that the mechanochemical reaction took place easily and could be attributed to the strong Lewis acid characteristic of FeCl_3 _[9] and the Lewis base characteristic of aniline, respectively. Although water was used to purify the product and could provide another possible reaction system for the polymerization, the washing time (several minutes) was obviously too short for the polymerization in the solution-based method.

On the formation of branched fibrous PANI, we believe that the mechanism is likely to be related to both the mechanochemical oxidation polymerization process and the linear nature of the PANI macromolecule chains [10]. It is known that the final structure of the products depends on the rates of nucleation and growth during the process of the reaction. At the start of the reaction, the aniline monomer is easily absorbed on the surface of treated CNFs through hydrogen bonding. When mixing with FeCl_3_, both the layered structure and strong Lewis acid characteristics of FeCl_3 _benefit the mechanochemical reaction with aniline as the NH_2 _group in aniline can easily coordinate to the iron ions with one free orbital through the free electron pair of the nitrogen atoms and complete the electron exchange [10]. Mechanical grinding not only causes new surfaces for the oxidation polymerization, but also promotes interfacial diffusion of the reactants and CNFs. Moreover, the diffusion of solid reactant particles at ambient temperature is often short-range during the mechanochemical reaction and is favorable for the formation of short polymer nanofibers. In addition, the *in situ *mechanochemical polymerization process may allow the polymerization to have a more site-selective interaction with the CNFs, resulting in a more effective electron delocalization and enhancing the electrochemical activity of the products. According to the conventional nucleation and growth theory [28], the PANI coating and the nanofibers that initially formed on the surfaces of CNFs serve as nucleation sites and surface active sites for further growth of the polymer shell and secondary fibers. Subsequent growth of PANI on such preformed rough polymer shells and nanofibers may lead to the formation of dendrites around the CNFs (as shown in Figure 7) since the heterogeneous growth of available nuclei is energetically more favorable than the formation of new nuclei [29].

## Conclusions

A facile process to functionalize carbon nanofiber with nanostructured polyaniline was developed via a simple mechanochemical *in situ *polymerization method. TEM examinations confirmed the grafting (and/or coating) of novel hierarchical nanostructured polyaniline onto carbon nanofibers. The resultant hybrid composites showed good dispersibility, and their dispersion had good stability which benefited their processability. Electrochemical tests also showed that the electrochemical specific capacitance of the PANI-functionalized CNFs was much larger than that of the CNFs.

## Abbreviations

CNF: carbon nanofiber; CNT: carbon nanotube; CV: cyclic voltammetry; DSC: differential scanning calorimetry; EB: emeraldine base; FT-IR: Fourier transform infrared; PANI: polyaniline; SEM: scanning electron microscopy; TEM: transmission electron microscopy; XRD: X-ray diffraction patterns.

## Competing interests

The authors declare that they have no competing interests.

## Authors' contributions

XD conceived the study, carried out most experiments and data analysis, and drafted the manuscript. GC and AB performed partial morphology analysis and were involved in revising the manuscript. H-YL and Y-WM were involved in the discussions and revisions of the manuscript. All authors read and approved the final manuscript.

## Supplementary Material

Additional file 1 **Xusheng Du NRL supporting information**. DTG curve of the treated CNF, SEM image of the PANI/CNF composites and CV of the PANI/CNF composites. Click here for file
