# Supplementary material for: Use of facile mechanochemical method to functionalize carbon nanofibers with nanostructured polyaniline and their electrochemical capacitance
Source: Nanoscale Res Lett. 2012 Feb 8;7(1):111. doi: 10.1186/1556-276X-7-111 (PMC3305476; doi:10.1186/1556-276X-7-111)
Supplement: Additional file 1 — Xusheng Du NRL supporting information. DTG curve of the treated CNF, SEM image of the PANI/CNF composites and CV of the PANI/CNF composites. [file 1556-276X-7-111-S1.DOC]

**Facile mechanochemical method to functionalize carbon nanofibers with nanostructured polyaniline and their electrochemical capacitance**

Xusheng Du*, Hong-Yuan Liu, Guipeng Cai, Yiu-Wing Mai*, Avinash Baji

Centre for Advanced Materials Technology (CAMT), School of Aerospace Mechanical & Mechatronic Engineering J07, University of Sydney, NSW 2006, Australia

**
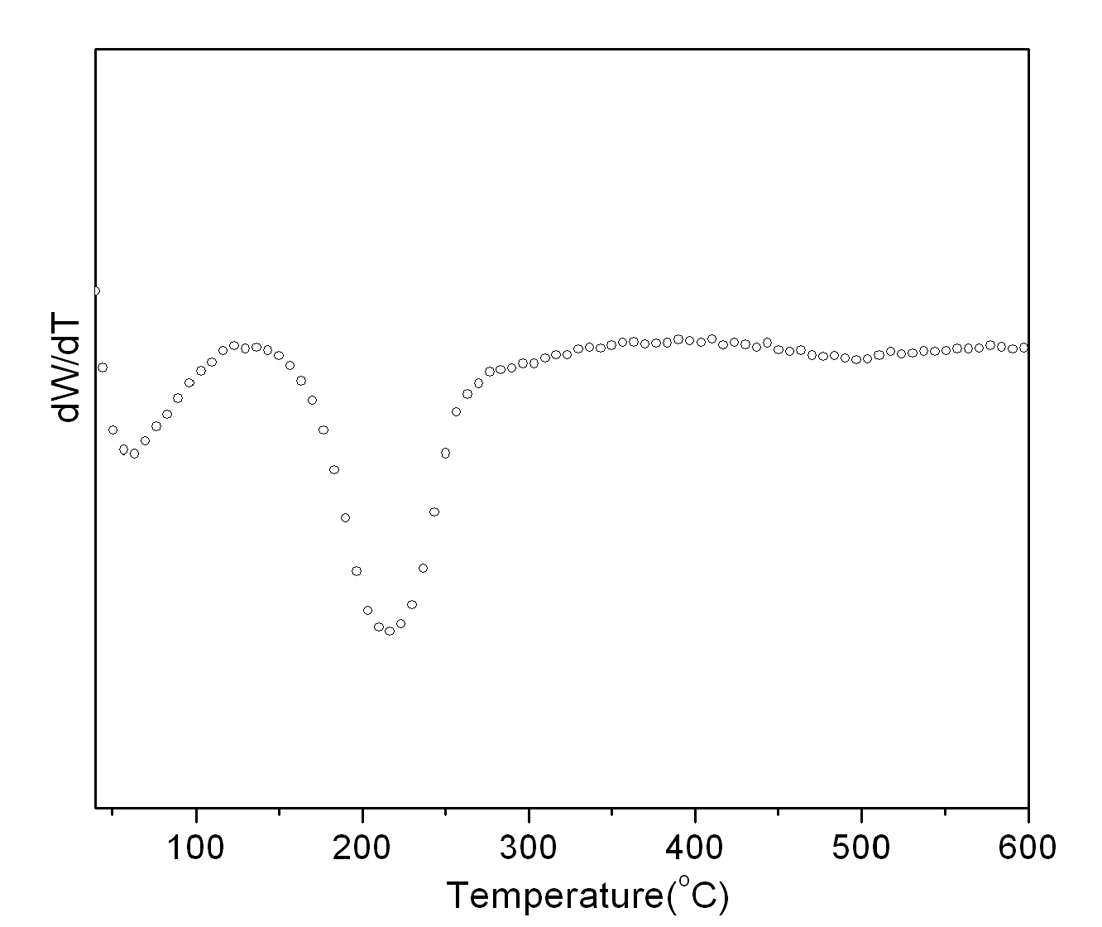
**

Figure S1. DTG curves of treated CNFs.


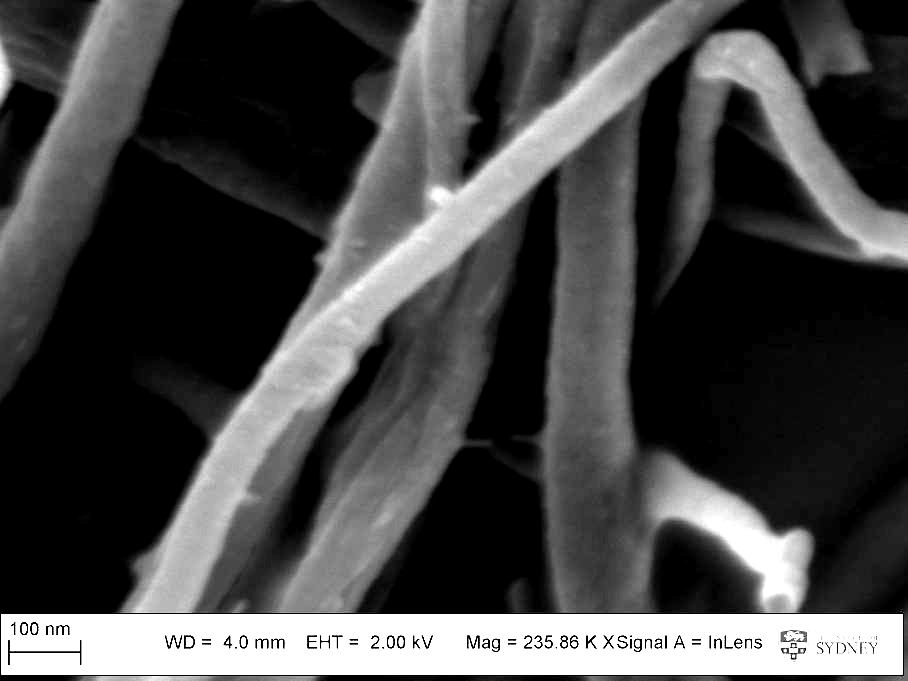


Figure S2. SEM image of PANI/CNF composite. Arrows highlight the nanostructured polyaniline on CNFs.

Figure S3. Cyclic voltammograms of PANI/CNF hybrid composite in 1M H_2_SO_4_ aqueous solution with a scan rate of 100 mV/s.
